# High co-occurrence of anorectal chlamydia with urogenital chlamydia in women visiting an STI clinic revealed by routine universal testing in an observational study; a recommendation towards a better anorectal chlamydia control in women

**DOI:** 10.1186/1471-2334-14-274

**Published:** 2014-05-19

**Authors:** Geneviève AFS van Liere, Christian JPA Hoebe, Petra FG Wolffs, Nicole HTM Dukers-Muijrers

**Affiliations:** 1 Department of Sexual Health, Infectious Diseases and Environmental Health, South Limburg Public Health Service, 6160, HA Geleen, Netherlands; 2 Department of Medical Microbiology, School of Public Health and Primary Care (CAPHRI), Maastricht University Medical Center (MUMC+), 6202, AZ Maastricht, Netherlands

**Keywords:** Anorectal, Urogenital, Women, Chlamydia, Gonorrhoea, Routine systematic testing, Control strategy, Treatment, Testing

## Abstract

**Background:**

Symptom- and sexual history-based testing i.e., testing on indication, for anorectal sexually transmitted infections (STIs) in women is common. Yet, it is unknown whether this strategy is effective. Moreover, little is known about alternative transmission routes i.e. by fingers/toys. This study assesses anorectal STI prevalence and infections missed by current testing practice, thereby informing the optimal control strategy for anorectal STIs in women.

**Methods:**

Women (n = 663) attending our STI-clinic between May 2012-July 2013 were offered routine testing for anorectal and urogenital *Chlamydia trachomatis* and *Neisseria gonorrhoeae.* Data were collected on demographics, sexual behaviour and symptoms. Women were assigned to one of the categories: indication (reported anal sex/symptoms), fingers/toys (only reported use of fingers/toys), or without indication.

**Results:**

Of women, 92% (n = 654) participated. There were 203 reports (31.0%) of anal sex and/or symptoms (indication), 48 reports (7.3%) of only using fingers/toys (fingers/toys), and 403 reports (61.6%) of no anal symptoms, no anal sex and no anal use of fingers/toys (without indication). The overall prevalence was 11.2% (73/654) for urogenital chlamydia and 8.4% (55/654) for anorectal chlamydia. Gonorrhoea infections were not observed. Prevalence of anorectal chlamydia was 7.9% (16/203) for women with indication and 8.6% (39/451) for all other women (P = 0.74). Two-thirds (39/55) of anorectal infections were diagnosed in women without indication. Isolated anorectal chlamydia was rare (n = 3): of all women with an anorectal infection, 94.5% (52/55) also had co-occurrence of urogenital chlamydia. Of all women with urogenital chlamydia, 71.2% (52/73) also had anorectal chlamydia.

**Conclusions:**

Current selective testing on indication of symptoms and sexual history is not an appropriate control strategy for anorectal chlamydia in women visiting an STI clinic. Routine universal anorectal testing is feasible and may be a possible control strategy in women. Yet costs may be a problem. When more restricted control measures are preferred, possible alternatives include (1) anorectal testing only in women with urogenital chlamydia (problem: treatment delay or loss to follow up), and (2) direct treatment for urogenital chlamydia that is effective for anorectal chlamydia as well.

## Background

*Chlamydia trachomatis* (Ct) and *Neisseria gonorrhoeae* (Ng) are the most prevalent bacterial sexually transmitted infections (STIs) in women in high income countries and have major public health consequences [1-3]. In addition to infection of the urogenital tract, chlamydia and gonorrhoea can also cause anorectal infections in women. Previous studies of women who visited an STI clinic or a department of genitourinary medicine found anorectal chlamydia in up to 18% [2,4-11] of them and gonorrhoea in up to 13% of them [4-10]. However, guidelines in UK, US and the Netherlands do not recommend routine anorectal testing, but restricted testing in people who are in high-risk groups, report anal sexual behaviour, or have anal symptoms [12], i.e., selective testing on indication [12,13]. This is in contrast to urogenital testing, which is a routine procedure in STI care services in these countries. Nucleic acid amplification tests (NAATs) are the most sensitive tests for the screening and diagnosis of genital chlamydial infections to date, and their use is accepted and recommended for anorectal infections as well [14]. The impact of anorectal infections in women on population (public health) and individual (clinical) level are yet unknown. However, it is suggested that treatment of anorectal infections in women can help limit the spread of STI in the population [4-6] and can reduce complications in infected individuals, such as anal cancer, anal squamous intraepithelial lesions [15,16] and reduce HIV risk [5,6]. Moreover, the rectum might act as a reservoir and thereby play a major role in repeat positive urogenital infections [4].

In the control of anorectal chlamydia there are 2 key stones: first is identification (diagnosis by testing) and second is treatment. In high risk groups there is evidence that many anorectal STI are missed by current testing practice on indication. A study using routine universal anorectal testing in high-risk women found that selective testing on indication misses over half of anorectal infections (48% Ct, 80% Ng) [17]. It is unknown whether selective testing on indication misses infections in the general female population, due to lack of studies in this population.

Adequate treatment for anorectal chlamydia is currently under debate. Guidelines in the UK and US recommend both single-dose azithromycin and a 7-day course of doxycycline as equal treatments for uncomplicated anorectal chlamydia in non pregnant women [12]. In the Netherlands, doxycycline is recommended for anorectal chlamydia [13]. Several studies have reported substantial microbial failure rates of up to 40% for single-dose azithromycin (1.0 g) used against anorectal chlamydia [18-22], or suggest that doxycycline may be more effective than azithromycin in the treatment of rectal chlamydial infections [23]. Anorectal control strategies (treatment and testing) are thereby in need of critical reflection.

To inform optimal control strategies for anorectal STIs in women, first the prevalence of anorectal STI was determined by using routine universal collected data in the general STI clinic population of women. Such data is scarce, because of the general lack of a routine universal screening practice in women in STI control settings. Moreover, little is known about alternative transmission routes such as the anal use of fingers and/or sex toys. By assessing associations with medical and behavioural history, we aim to estimate the number of anorectal infections missed by the current practice of selective testing on indication and to formulate recommendations for control i.e., the testing and treatment strategies.

## Methods

### Study population

The outpatient STI clinic of the South Limburg Public Health Service provides about 6000 consultations annually, offering free examination and treatment at three regional outpatient STI clinics. Between May 2012 and July 2013, three consultation nurses (out of 13) offered all their female patients aged 18 years and older (n = 663) routine testing for urogenital and anorectal chlamydia and gonorrhoea. This yielded a total of 654 consultations by 611 women for analysis (participation 92.2%). Fifty-two (7.8%) women declined an anorectal swab; reported reasons for non-participation were inconvenience (65%), fear (19%) and lack of necessity (16%). Non-participants were slightly younger than participants (median 21 years versus 23 years, P < 0.001). Urogenital chlamydia prevalence was similar for non-participants and participants (13.5%; (7/52) versus 11.2% (73/654), P = 0.60). Gonorrhoea infections were not observed in both groups. The study was approved by the Medical Ethics Committee of Maastricht University (11-4-108).

### Study procedures and definitions

Women provided self-collected vaginal swabs and self-collected anorectal swabs, which studies have proven to be a generally acceptable, valid and feasible approach [3,24,25]. Trained study nurses provided women with a visual diagram and oral instructions about how to take separate self-collected vaginal and rectal swabs. For the vaginal swab, the patient was instructed to insert the swab 2.5 cm into the vagina, rotate it for 5 to 10 seconds, and then place it in a capped tube to avoid potential contamination. This procedure was repeated in the anus for the rectal swab. Samples were tested for *Chlamydia trachomatis* and *Neisseria gonorrhoeae* using nucleic acid amplification assays according to the manufacturer’s procedure (polymerase chain reaction [PCR; Roche Cobas 4800, San Francisco, CA]). Serum was tested for Treponema pallidum hemagglutination (TPHA) and HIV; all the women were TPHA and HIV negative. Each consult also included a standardised medical and sexual history taken by trained study nurses. It asked about self-reported symptoms and sexual behaviour in the past six months, i.e., ‘Did you practise anal sex in the past six months?’. Anal symptoms included rectal discharge, bleeding, pain, redness, burning sensation, or itching. Swingers were defined as women who were part of a male–female couple that had sex with other male–female couples and their self-identified heterosexual sex partners. Prostitutes were defined as women who reported having had sex for money in the past six months. Women who were prostitute and/or swinger were defined as prostitutes/swingers. All data was registered in an electronic patient registry.

### Statistical analysis

Women were assigned to one of three non-overlapping hierarchically constructed indication categories based on reported behaviour and symptoms. Women in the “indication” category reported at least anal symptoms and/or anal sex, whether or not in combination with anal use of fingers and/or toys. Women who were assigned to the “fingers/toys” category only reported the anal use of fingers and/or toys and reported no anal symptoms and no anal sex. Women who reported no anal symptoms, no anal sex, and no anal use of fingers or toys were assigned to the “without indication” category. As no gonorrhoea infections were observed, statistical analyses focussed on chlamydia only. The prevalence of chlamydia was calculated by dividing the number of positive tests by the total number of tests, multiplied by 100. Univariate and multivariate logistic regression were used to identify determinants independently associated with anorectal chlamydia. Determinants tested were indication (with indication versus the two other categories combined), age categories, prostitutes/swingers (prostitutes and swingers versus other women), and use of fingers/toys (versus no use of fingers/toys). Anorectal infections in the categories “without indication” and “fingers/toys” were defined as infections missed by selective testing on indication as in current care. The share of infections missed was compared between indication categories, age categories (reference ≥ 29 years) and prostitutes/swingers using univariate and multivariate logistic regression. Interactions terms were added between indication categories, age categories and prostitutes/swingers in the multivariate models, but none were statistically significant and were removed from in the final models.

Finally, to assess the anatomic site distributions of urogenital and anorectal chlamydia, all women who tested positive for chlamydia were assigned to a non-overlapping distribution category: (1) urogenital only, (2) urogenital and anorectal, or (3) anorectal only. Restricting to chlamydia positives, this variable was compared over indication categories (with indication versus the two other categories combined), age categories, and prostitutes/swingers using Fisher’s exact test. A P value of < 0.05 was considered statistically significant. Analyses were performed using SPSS version 17.0.0 (IBM Inc., Somers, NY, USA). Written informed consent for participation in the study was obtained from participants. Written informed consent was not obtained from a parent or guardian.

## Results

We included 654 consultations with an overall median participant age of 23 years (inter-quartile range: 21 to 34). The overall prevalence was 11.2% (73/654) for urogenital chlamydia and 8.4% (55/654) for anorectal chlamydia. Gonorrhoea infections were not observed. Overall, anal use of fingers was reported by 20.3% (133/654) of the women, anal use of toys by 8.9% (58/654), anal sex with a steady partner by 24.0% (157/654), anal sex with a casual partner by 13.1% (86/654) and anal symptoms by 3.1% (20/654). Anal symptoms reported were itching (n = 7), ulceration (n = 3), redness (n = 2), discharge (n = 1), pain/burning sensation (n = 5), bleeding (n = 3) and unspecified (n = 2). Only 3 women reported a combination of (two) symptoms.

### Indication categories

In total, 31.0% (203/654) of the women were assigned to the “indication” category (i.e., they reported anal sex and/or symptoms), 7.3% (48/654) to the “fingers/toys” category (reported anal use of fingers/toys only), and 61.6% (403/654) to the “without indication” category (no reported anal symptoms, no anal sex, and no anal use of fingers/toys). Women without indication were younger than women with indication (median of 22 years versus 36 years). The share of prostitutes/swingers was higher in women using fingers/toys only compared to women without indication (Table 1).

**Table 1:** Characteristics of women attending the STI clinic routinely screened for urogenital and anorectal chlamydia

	**Indication**^**#**^	**Fingers/toys only**	**Without indication**	**Total**
	**N = 203**	**N = 48**	**N = 403**	**N = 654**
	**% (n)**	**% (n)**	**% (n)**	**% (n)**
Age				
≤ 21 years	19.2 (39)**	12.5 (6)**	41.2 (166)	32.3 (211)
22-28 years	27.1 (55)	25.0 (12)	40.0 (161)	34.9 (228)
≥ 29 years	53.7 (109)	62.5 (30)	18.9 (76)	32.9 (215)
Prostitutes/swingers	39.4 (80)*	66.7 (32)*	13.9 (56)	25.7 (168)
Chlamydia prevalence				
Urogenital	9.4 (19)	4.2 (2)	12.9 (52)	11.2 (73)
Anorectal	7.9 (16)	4.2 (2)	9.2 (37)	8.4 (55)
Anatomic site distribution chlamydia positives	N = 21	N = 2	N = 53	N = 76
Urogenital only	23.8 (5)	0 (0)	30.2 (16)	27.6 (21)
Anorectal only	9.5 (2)	0 (0)	1.9 (1)	3.9 (3)
Urogenital and anorectal	66.7 (14)	100 (2)	67.9 (36)	68.4 (52)

*P < 0.0001 compared to without indication by Chi square test.

**P < 0.0001 compared to without indication and age 29 years or older by Chi square test.

^#^Women with indication reported anal use of fingers and/or toys in 43.3% of consultations, symptoms in 9.9%, anal sex with a steady partner in 77.3%, and anal sex with a casual partner in 42.4%.

### Chlamydia prevalence and associated determinants

Prevalence of anorectal chlamydia was 7.9% (16/203) for women with indication and 8.6% (39/451) for the other women (categories without indication and fingers/toys) (P = 0.74). Prevalence in the three indication categories is displayed in Table 1. Young age was the only determinant associated with anorectal chlamydia (≤ 21 years 14.2% (odds ratio 3.79 (1.75-8.20)), 22–28 years 7.0% (odds ratio 1.73 (0.75-4.00) and ≥ 29 years 4.2%). Being prostitute/swinger was not associated with anorectal chlamydia; prevalence was 3.0% (5/168) for prostitutes/swingers versus 10.3% (50/486) for other women (P = 0.13). In total, 136 women reported to have used fingers or toys, whether or not in combination with anal sex. Prevalence in those women was 5.1% (7/136) versus 9.3% (48/518) in women who did not report to have used fingers or toys (P = 0.82).

### Missed infections by selective testing on indication

In total, 55 anorectal chlamydia infections were diagnosed; 67.3% (37/55) were diagnosed in women without indication (Table 1). Only 2/55 anorectal infections were diagnosed in the fingers/toys category. Combining fingers/toys with the without indication category (as is usually the case in current care), the proportion missed by current care that uses selective testing was 70.9% (39/55). No determinants were found to be associated with missed anorectal infections. For example the proportion missed was 60.0% in prostitutes/swingers versus 72.0% in other women (P = 0.93). The proportion missed was 70.0% in age ≤ 21 years, 81.3% in age 22–28 years and 55.6% in age ≥ 29 years (P = 0.47).

### Anatomic site distribution

**Figure 1:**
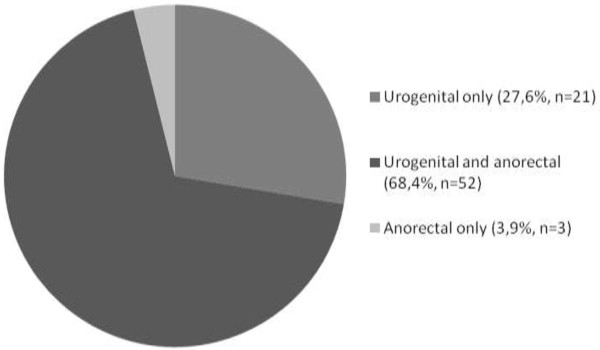
Anatomic site distribution of chlamydia in women attending the STI clinic routinely screened for urogenital and anorectal chlamydia.

Of all urogenital and anorectal chlamydia infections found, 68.4% (52/76) were concurrent urogenital and anorectal infections. Of all chlamydia infections, only three infections were isolated anorectal (3.9%, 3/76): two in women with indication and one in a woman without indication (Figure 1). Of the 73 women with urogenital chlamydia, 71.2% (52/73) also had an anorectal chlamydia infection. Of the 55 women with anorectal chlamydia, 94.5% (52/55) also had a urogenital chlamydia infection. The anatomic site distribution of chlamydia infections was not associated with indication categories (P = 0.31), age (P = 0.90), or prostitutes/swingers (P = 0.27) (Table 1).

## Discussion

This study revealed alarmingly high numbers of anorectal chlamydia in women. One in 12 women and even one in 7 young women was diagnosed with anorectal chlamydia. As over two thirds of these infections are currently being missed, current selective testing on indication of symptoms and anal sexual history is not an appropriate control strategy for anorectal chlamydia in women visiting an STI clinic. Almost all women with anorectal chlamydia had concurrent urogenital chlamydia (95%). To our knowledge, this is the first study with routine universal anorectal testing, i.e., independent of reported behaviour, symptoms or urogenital positivity, in a general group of women who visited an STI clinic and who took different sexual risks, including anal use of fingers and toys.

Indication (anal sex or symptoms) was not associated with anorectal chlamydia. Yet current guidelines for anorectal STIs advocate selective symptom- and sexual history based testing for women [12,13]. Including the anal use of fingers/toys in this testing on indication would only reveal a small number of missed infections (3.6%), as these practices are common but rarely practiced without anal sex (7%). Moreover, use of fingers/toys was not associated with anorectal chlamydia. This suggests that anal use of fingers/toys, as well as report of anal sex or symptoms are not useful indicators to guide testing for anorectal chlamydia.

The prevalence of anorectal chlamydia was substantial (8%) (55/654). Young age (≤ 21 years) was found to be associated with anorectal chlamydia showing a strikingly high 14% prevalence in this group of women, consistent with studies in high risk women visiting an STI clinic [5,6,9]. Thereby the absolute number of anorectal infections that are currently missed in the general female STI clinic population is likely substantial, especially in young women. This is consistent with earlier findings in high-risk groups at our clinic, such as female swingers (chlamydia 48%, gonorrhoea 80%) and men who have sex with men (MSM) (chlamydia 43-55%, gonorrhoea 29-100%) [17]. More data is needed from other settings to confirm our observations by routine anorectal testing.

In contrast to MSM, anorectal chlamydia in women was rarely isolated. In current study, one woman had an isolated anorectal infection but did not report anal sex or symptoms. Possible explanations for this could be underreporting, a false negative urogenital test [4,8], or autoinoculation from a spontaneously cleared urethral/vaginal infection [4,5]. The large percentage of concurrent urogenital and anorectal chlamydia infections in women was therefore notable: 95% of women with anorectal chlamydia also had urogenital chlamydia, and 71% of women with urogenital chlamydia also had anorectal chlamydia. Previous studies without routine universal testing in women also reported large shares of concurrent infections (36-90%) [2,4-10,26]. It is not clear what causes these concurrent infections, although possible explanations could be underreporting of anal sex, autoinoculation with vaginal secretions [4,5,8,9,26] or concurrent transmission during sex. Majority (71%) of anorectal chlamydia positives did not report anal sex or symptoms. Autoinoculation from the vagina to the rectum therefore seems possible. We hypothesize that autoinoculation could also occur from the rectum to the vagina. Even in the absence of sexual activity, the gastro intestinal tract could provide a constant source of organisms which may reinfect the genital tract [27]. Such (repeat) urogenital infections could lead to reproductive tract morbidity [6]. Further study on this subject is needed, for example by including anorectal chlamydia in mathematical models and by bacterial load studies, the clinical and public health impact of anorectal chlamydia in women could be explored further.

Nevertheless, state of the art practice in chlamydia control entails the use of highly sensitive NAATs to test for chlamydia. Although NAATs are not yet FDA proved for anorectal testing, their use is highly recommended, accepted, and part of standard operating procedures in many care settings [14]. A positive NAAT, i.e., diagnosed anorectal chlamydia, is in practice followed by antibiotic treatment. In MSM, an anorectal swab positive for chlamydia is considered an infection, and is treated with antibiotics to prevent transmission to the population and complications in individuals. To overcome current insufficient case management of anorectal infections in women, testing and treatment strategies need to be improved, to better identify and treat infections. Study participation was high (93%), suggesting a high feasibility and acceptability of anorectal testing in women who do not have an indication. Therefore, routine universal anorectal screening could be an option, although this will increase costs substantially. No studies have evaluated cost effectiveness of anorectal screening for chlamydia/gonorrhoea in women. However, in MSM, anorectal screening (when prevalence > 2.69% (IQR, 1.68-3.71%)) can be a cost-effective intervention to reduce HIV infection [28-30].

When a more restricted policy is preferred, anorectal testing only in women with urogenital infection or direct treatment effective for both urogenital and non-urogenital chlamydia would detect and treat 95% of anorectal infections, since 52 of 55 anorectal infections had co-occurrence of urogenital chlamydia. However, for the former option delay between urogenital and anorectal tests and subsequent treatments could be a problem in practice. The substantial anorectal chlamydia prevalence and high co-occurrence with urogenital chlamydia fuels the need for debate on what is adequate treatment for anorectal chlamydia [18-21,23]. The currently used treatment regimes for uncomplicated anorectal chlamydia both have drawbacks; higher treatment failure rates are reported for azithromycin [19-21,23] and compliance for doxycycline could possibly be an issue in practice [31]. More research, for example a randomised controlled trial of azithromycin versus doxycycline, including compliance, is needed to formulate treatment recommendations.

Several study limitations need to be acknowledged. We only included women who visited the STI clinic, so our results might not fully represent those that could be found in the general female population or within other healthcare settings. Although our instructions on specimen collection were clear, we cannot entirely rule out the possibility of specimen contamination (e.g., via the urogenital-anorectal route). As women attending the STI clinic were randomly assigned to a consultation nurse, possible selection bias is likely minimal. Data on other high risk sexual behaviors (i.e., number of partners, new or concurrent partnerships, substance use, condom use) were not available, and their association with anorectal chlamydia in women could not be assessed. In our study, the prevalence or the proportion of infections missed by current selective testing in the non-participants is unknown. Eligible non-participants were slightly younger than participants. A study by Sethupathi et al. found women most at risk for anorectal infections included women aged < 20 years as was also found in current study. Therefore, the prevalence of anorectal infections may be underestimated in current study, yet due to the high response (93%), bias is expected to be minimal.

## Conclusions

In conclusion, prevalence of anorectal chlamydia in women was high and current selective testing on indication is not an appropriate control strategy to identify and treat anorectal chlamydia infections. Almost all women with anorectal chlamydia had concurrent urogenital chlamydia. More research is needed on the public health and clinical implications of anorectal chlamydia in women.

## Competing interests

All authors have completed the ICMJE uniform disclosure form at http://www.icmje.org/coi_disclosure.pdf (available upon request from the corresponding author) and declare the following: no support from any organisation for the submitted work; no financial relationships with any organisations that might have an interest in the submitted work in the previous three years; no other relationships or activities that could appear to have influenced the submitted work.

## Authors’ contributions

CJPAH and NHTMDM were involved in the conception and design of the study. GAFSvL produced the database, analysed the data and wrote the first draft of the manuscript. All of the authors contributed to writing the paper; all had full access to all of the data in the study, and all can take responsibility for the integrity of the data and the accuracy of the data analysis. All authors read and approved the final manuscript.

## Pre-publication history

The pre-publication history for this paper can be accessed here:

http://www.biomedcentral.com/1471-2334/14/274/prepub
